# Supplementary material for: A Single-Level Tunnel Model to Account for Electrical Transport through Single Molecule- and Self-Assembled Monolayer-based Junctions
Source: Sci Rep. 2016 May 24;6:26517. doi: 10.1038/srep26517 (PMC4877922; doi:10.1038/srep26517)
Supplement: Supplementary Information [file srep26517-s1.pdf]

Supplemental Information

A Single-Level Tunnel Model to Account for Electrical Transport through Single Molecule- and Self-Assembled Monolayer-based Junctions

by

A. R. Garrigues, L. Yuan, L. Wang, E. R. Mucciolo, D. Thompson, E. del Barco, and C. A. Nijhuis

1 The rate equation formulation

Let M be the number of spin-split orbitals in the molecule. The number of possible electronic configurations is equal to $n = 2^M$ (i.e., each orbital can be empty or occupied). Each configuration can be described by a set of occupation numbers $c_\alpha(i)$, with $\alpha = 1, \ldots, n$ and $i = 1, \ldots, M$, where

$$c_\alpha(i) = 0 \,\mathrm{or}\, 1, \tag{1}$$

depending on whether the i-th orbital is occupied or not. Call P_α the probability of the α configuration, with $P_\alpha \geq 0$ and $\sum_{\alpha=1}^n P_\alpha = 1$.

Under certain approximations, which neglect memory effects and discard correlations between the electrodes and the molecule, the rate equation governing the change in the configuration probabilities over time can be expressed as [1,2,3]

$$\frac{dP_\alpha}{dt} = -P_\alpha \sum_{\beta \neq \alpha} \Gamma_{\alpha \to \beta} + \sum_{\beta \neq \alpha} \Gamma_{\beta \to \alpha} P_\beta, \tag{2}$$

where $\Gamma_{\alpha \to \beta}$ is the rate of the $\alpha \to \beta$ transition. Equation (2) is the well-known Pauli master equation [4]. We note here that there are several ways to derive this equation from fundamental theories, such as nonequilibrium quantum statistical mechanics [5,6,7], as well as from a semiclassical Boltzmann kinetic equation [1]. Here, rather than repeating those standard derivations, we focus on the use of the rate equation to compute transport proeperties in the context of the experiments reported in the main text. The main goal is to obtain expressions for the charge current across the molecule in different asymptotic regimes.

We can express Eq. (2) in matrix form,

$$\frac{dP_\alpha}{dt} = \sum_\beta \Lambda_{\alpha\beta} P_\beta, \tag{3}$$

where

$$\Lambda_{\alpha\beta} = \begin{cases} -\sum_{\gamma \neq \alpha} \Gamma_{\alpha \to \gamma}, \mathrm{if}\ \alpha = \beta \\ \Gamma_{\beta \to \alpha}, \mathrm{if}\ \alpha \neq \beta \end{cases}. \tag{4}$$

Notice that, consistent with the normalization condition, we have

$$\begin{aligned} \sum_{\alpha=1}^n \frac{dP_\alpha}{dt} &= -\sum_{\alpha=1}^n P_\alpha \sum_{\beta \neq \alpha} \Gamma_{\alpha \to \beta} + \sum_{\alpha=1}^n \sum_{\beta \neq \alpha} \Gamma_{\beta \to \alpha} P_\beta && (5) \\ &= -\sum_{\alpha=1}^n P_\alpha \sum_{\beta \neq \alpha} \Gamma_{\alpha \to \beta} + \sum_{\beta=1}^n P_\beta \sum_{\alpha \neq \beta} \Gamma_{\beta \to \alpha} && (6) \\ &= 0, && (7) \end{aligned}$$

implying that

$$\sum_{\alpha=1}^n \Lambda_{\alpha\beta} = 0, \tag{8}$$

as it can be easily verified. On the other hand,

$$\begin{aligned} \sum_{\beta=1}^n \Lambda_{\alpha\beta} &= -\sum_{\gamma \neq \alpha} \Gamma_{\alpha \to \gamma} + \sum_{\beta \neq \alpha} \Gamma_{\beta \to \alpha} && (9) \\ &= \sum_{\beta \neq \alpha} (\Gamma_{\beta \to \alpha} - \Gamma_{\alpha \to \beta}). && (10) \end{aligned}$$

Thus, if $\Gamma_{\beta\to\alpha} = \Gamma_{\alpha\to\beta}$ for all transitions, then the r.h.s. of this equation is equal to zero. This means that a trivial stationary solution exits where $P_\alpha = 1/n$ for all $\alpha = 1, ..., n$. However, when $\Gamma_{\beta\to\alpha} \neq \Gamma_{\alpha\to\beta}$, other nontrivial stationary solution exist as well.

2 Stationary solutions

In order to obtain a stationary solution to the rate equations set

$$\frac{dP_\alpha}{dt} = 0 \tag{11}$$

for all $\alpha = 1, ..., n$. This implies

$$\sum_\beta \Lambda_{\alpha\beta} P_\beta = 0. \tag{12}$$

Thus, to find the set of stationary probabilities $\{P_\alpha\}$, one needs to find the right eigenvector corresponding to the zero eigenvalue of the matrix Λ.

3 Transition rates

Let E_α be the total energy of the molecule and N_α be the total number of electrons in the α configuration. When an electron hops from one of the leads and into the molecule, energy conservation requires

$$\varepsilon + E_\alpha = E_\beta, \tag{13}$$

where $\alpha(\beta)$ is the molecule's configuration before(after) the hopping and ε is the energy of the electronic state in the lead. For this transition to take place, the state with energy ε in the lead must have a finite occupation number, namely $f\left(\frac{\varepsilon - \mu_l}{k_B T}\right) > 0$, where μ_l is the lead's chemical potential ($l = R, L$) and T is the temperature. Here, $f(x)$ denotes the Fermi-Dirac distribution,

$$f(x) = \frac{1}{e^x + 1}. \tag{14}$$

In the opposite case, when an electron hops from the molecule and into the lead, we have

$$E_\alpha = \varepsilon + E_\beta. \tag{15}$$

Now, the occupation number of the state with energy ε must be such that $f\left(\frac{\varepsilon - \mu_l}{k_B T}\right) < 1$.

Call γ_R and γ_L the level widths due to the coupling to the right and left leads, respectively. We can split the transition rate into two contributions,

$$\Gamma_{\alpha\to\beta} = \Gamma^R_{\alpha\to\beta} + \Gamma^L_{\alpha\to\beta}, \tag{16}$$

where [1,2,3]

$$\Gamma^l_{\alpha\to\beta} = \begin{cases} (\gamma_l/\hbar)\, f\left(\frac{E_\beta - E_\alpha - \mu_l}{k_B T}\right), \text{if } N_\beta = N_\alpha + 1 \text{ and } d_H(c_\beta, c_\alpha) = 1 \\ (\gamma_l/\hbar)\left[1 - f\left(\frac{E_\alpha - E_\beta - \mu_l}{k_B T}\right)\right], \text{if } N_\beta = N_\alpha - 1 \text{ and } d_H(c_\beta, c_\alpha) = 1 \\ 0, \text{otherwise} \end{cases}, l = R, L, \tag{17}$$

where $d_H(c_\beta, c_\alpha)$ is the Hamming distance between the binary sets c_β and c_α. Namely, only transitions where the number of electrons in the molecule changes by one are allowed. Notice that the bias voltage is equal to $V = (\mu_L - \mu_R)/e$, where e denotes the electron charge. Equation (17) can be derived in a number of ways, with the most standard being Fermi's Golden Rule or time-dependent perturbation theory on the level widths γ_R and γ_L.

The rate equation approach is valid when $k_B T \gg \gamma_{R,L}$ (since it assumes a perfectly sharp energy lever in the molecule), and when the tunneling through the molecule is sequentially incoherent. Below, we discuss the validity of the rate equation in more detail.

Implicit in Eq. (17) is the assumption that the molecule's energy levels are sharp, such that γ_R, γ_L are much smaller than other energy scales of the problem, such as $k_B T$, eV, and the separation of energy levels in the molecule.

4 Current

The current coming from the left lead is equal to

$$I_L = -e \sum_{\alpha,\beta} \Delta N_{\alpha\to\beta} \Gamma^L_{\alpha\to\beta} P_\alpha, \tag{18}$$

where $\Delta N_{\alpha\to\beta} = N_\beta - N_\alpha$.

5 Total energy

The total energy in the molecule can be broken down as follows (constant charging energy model):

$$E_\alpha = \frac{1}{2} N_\alpha (N_\alpha - 1) E_c - e V_g N_\alpha + \sum_{i=1}^{M} c_\alpha(i)\, \varepsilon_i, \tag{19}$$

where E_c is the charging energy, V_g is the gate voltage, and $\{\varepsilon_i\}_{i=1,\ldots,M}$ are the energies of the orbitals.

6 Single-level case

Let us apply this formulation compute the stationary current of a molecule with a single orbital (spinless case), in which case $M = 1$ and $n = 2$. $P_{0(1)}$ corresponds to the probability of the empty(filled) state. The stationary problem is defined by the matrix

$$\Lambda = \begin{pmatrix} -\Gamma_{0\to1} & \Gamma_{1\to0} \\ \Gamma_{0\to1} & -\Gamma_{1\to0} \end{pmatrix}, \tag{20}$$

where

$$\Gamma_{0\to1} = \Gamma^R_{0\to1} + \Gamma^L_{0\to1} \tag{21}$$

and

$$\Gamma_{1\to0} = \Gamma^R_{1\to0} + \Gamma^L_{1\to0}\,, \tag{22}$$

with

$$\Gamma^l_{0\to1} = (\gamma_l/\hbar)\, f\!\left(\frac{E_1 - E_0 - \mu_l}{k_B T}\right), l = L, R, \tag{23}$$

and

$$\Gamma^l_{1\to0} = (\gamma_l/\hbar) \left[1 - f\!\left(\frac{E_1 - E_0 - \mu_l}{k_B T}\right)\right], l = L, R. \tag{24}$$

The eigenvector of the Λ matrix with zero eigenvalue corresponds to

$$P_0 = \frac{\Gamma_{1\to0}}{\Gamma_{1\to0} + \Gamma_{0\to1}} = \frac{\hbar\Gamma_{1\to0}}{\gamma_R + \gamma_L} \tag{25}$$

and

$$P_1 = \frac{\Gamma_{0\to1}}{\Gamma_{1\to0} + \Gamma_{0\to1}} = \frac{\hbar\Gamma_{0\to1}}{\gamma_R + \gamma_L}. \tag{26}$$

The current coming from the left lead is equal to

$$
\begin{aligned}
I_L &= -e\,(P_0\,\Gamma^L_{0\to1} - P_1\,\Gamma^L_{1\to0}) && (27)\\
&= -\frac{e\hbar}{\gamma_R+\gamma_L}\,(\Gamma_{1\to0}\,\Gamma^L_{0\to1} - \Gamma_{0\to1}\,\Gamma^L_{1\to0}) && (28)\\
&= -\frac{e\hbar}{\gamma_R+\gamma_L}\,(\Gamma^R_{1\to0}\,\Gamma^L_{0\to1} - \Gamma^R_{0\to1}\,\Gamma^L_{1\to0}) && (29)\\
&= -\frac{e}{\hbar}\frac{\gamma_R\gamma_L}{\gamma_R+\gamma_L}\left\{\left[1-f\left(\frac{E_{10}-\mu_R}{k_BT}\right)\right]f\left(\frac{E_{10}-\mu_L}{k_BT}\right) - f\left(\frac{E_{10}-\mu_R}{k_BT}\right)\left[1-f\left(\frac{E_{10}-\mu_L}{k_BT}\right)\right]\right\} && (30)\\
&= -\frac{e}{\hbar}\frac{\gamma_R\gamma_L}{\gamma_R+\gamma_L}\left[f\left(\frac{E_{10}-\mu_L}{k_BT}\right) - f\left(\frac{E_{10}-\mu_R}{k_BT}\right)\right], && (31)
\end{aligned}
$$

where $E_{10} = E_1 - E_0 = -eV_g + \varepsilon_1$.

6.1 Finite level width

When the broadening of the energy level is not negligible, we have to modify the calculations to account for the uncertainty in ϵ_1. Let γ be the total level width and $D_1(\varepsilon)$ the density of state profile associated to the single-level configuration; for instance, consider the Lorentzian profile

$$D_1(\varepsilon) = \frac{1}{\pi}\frac{\gamma/2}{(\varepsilon-\varepsilon_1)^2 + (\gamma/2)^2}, \tag{32}$$

with $\int\, d\varepsilon\, D_1(\varepsilon) = 1$. The modified expressions for the transition rates are

$$\Gamma^l_{0\to1} = (\gamma_l/\hbar)\int d\varepsilon\, D_1(\varepsilon)\, f\left(\frac{E_1-E_0-\mu_l}{k_BT}\right), l = L, R, \tag{33}$$

and

$$\Gamma^l_{1\to0} = (\gamma_l/\hbar)\int d\varepsilon\, D_1(\epsilon)\left[1-f\left(\frac{E_1-E_0-\mu_l}{k_BT}\right)\right], l = L, R, \tag{34}$$

where

$$E_1 - E_0 = -eV_g + \varepsilon. \tag{35}$$

Notice that

$$\Gamma^R_{0\to1} + \Gamma^R_{1\to0} = (\gamma_R/\hbar)\int d\varepsilon\, D_1(\varepsilon) = \gamma_R/\hbar \tag{36}$$

and

$$\Gamma^L_{0\to1} + \Gamma^L_{1\to0} = (\gamma_L/\hbar)\int d\varepsilon\, D_1(\varepsilon) = \gamma_L/\hbar. \tag{37}$$

Therefore,

$$\Gamma_{0\to1} + \Gamma_{1\to0} = (\gamma_R+\gamma_L)/\hbar, \tag{38}$$

$$
\begin{aligned}
P_0 &= \frac{1}{\gamma_R+\gamma_L}\int d\varepsilon\, D_1(\varepsilon)\sum_l \gamma_l\left[1-f\left(\frac{E_1-E_0-\mu_l}{k_BT}\right)\right] && (39)\\
&= \frac{1}{\gamma_R+\gamma_L}\sum_l \gamma_l\int d\epsilon\varepsilon D_1(\varepsilon+eV_g)\left[1-f\left(\frac{\varepsilon-\mu_l}{k_BT}\right)\right] && (40)
\end{aligned}
$$

and

$$
\begin{aligned}
P_1 &= \frac{1}{\gamma_R+\gamma_L}\int d\varepsilon\, D_1(\varepsilon)\sum_l \gamma_l\, f\left(\frac{E_1-E_0-\mu_l}{k_BT}\right) && (41)\\
&= \frac{1}{\gamma_R+\gamma_L}\sum_l \gamma_l\int d\varepsilon\, D_1(\varepsilon+eV_g)\, f\left(\frac{\varepsilon-\mu_l}{k_BT}\right). && (42)
\end{aligned}
$$

Going back to the expression defining the current through the left lead, we find

$$I_L = -e\,(P_0\,\Gamma^L_{0\to1} - P_1\,\Gamma^L_{1\to0}) \tag{43}$$

$$= -\frac{e}{\hbar}\frac{1}{\gamma_R+\gamma_L}(\Gamma_{1\to0}\,\Gamma^L_{0\to1} - \Gamma_{0\to1}\,\Gamma^L_{1\to0}) \tag{44}$$

$$= -\frac{e}{\hbar}\frac{1}{\gamma_R+\gamma_L}[(\Gamma^R_{1\to0}+\Gamma^L_{1\to0})\,\Gamma^L_{0\to1} - (\Gamma^R_{0\to1}+\Gamma^L_{0\to1})\,\Gamma^L_{1\to0}] \tag{45}$$

$$= -\frac{e}{\hbar}\frac{1}{\gamma_R+\gamma_L}[\Gamma^R_{1\to0}\,\Gamma^L_{0\to1} - \Gamma^R_{0\to1}\,\Gamma^L_{1\to0}] \tag{46}$$

$$= -\frac{e}{\hbar}\frac{\gamma_R\gamma_L}{\gamma_R+\gamma_L}\int d\varepsilon\,D_1(\varepsilon)\int d\varepsilon'\;D_1(\varepsilon')\left\{\left[1 - f\left(\frac{-eV_g+\varepsilon-\mu_R}{k_BT}\right)\right]f\left(\frac{-eV_g+\varepsilon'-\mu_L}{k_BT}\right) - f\left(\frac{-eV_g+\varepsilon-\mu_R}{k_BT}\right)\left[1-f\left(\frac{-eV_g+\varepsilon'-\mu_L}{k_BT}\right)\right]\right\} \tag{47}$$

$$= -\frac{e}{\hbar}\frac{\gamma_R\gamma_L}{\gamma_R+\gamma_L}\int d\varepsilon\,D_1(\varepsilon)\int d\varepsilon'\,D_1(\varepsilon')\left\{f\left(\frac{-eV_g+\varepsilon'-\mu_L}{k_BT}\right) - f\left(\frac{-eV_g+\varepsilon-\mu_R}{k_BT}\right)\right\} \tag{48}$$

$$= -\frac{e}{\hbar}\frac{\gamma_R\gamma_L}{\gamma_R+\gamma_L}\int d\varepsilon\,D_1(\varepsilon)\left[f\left(\frac{-eV_g+\varepsilon-\mu_L}{k_BT}\right) - f\left(\frac{-eV_g+\varepsilon-\mu_R}{k_BT}\right)\right] \tag{49}$$

$$= -\frac{e}{\hbar}\frac{\gamma_R\gamma_L}{\gamma_R+\gamma_L}\int d\varepsilon\,D_1(\varepsilon+eV_g)\left[f\left(\frac{\varepsilon-\mu_L}{k_BT}\right) - f\left(\frac{\varepsilon-\mu_R}{k_BT}\right)\right] \tag{50}$$

$$= -\frac{e}{\hbar}\frac{\gamma_R\gamma_L}{\gamma_R+\gamma_L}\int d\varepsilon\,\frac{\gamma}{(\varepsilon-\varepsilon_1+eV_g)^2+(\gamma/2)^2}\left[f\left(\frac{\varepsilon-\mu_L}{k_BT}\right) - f\left(\frac{\varepsilon-\mu_R}{k_BT}\right)\right]. \tag{51}$$

This expression generalizes the previous one, Eq. (31), to include a finite level width γ. It is straightforward to check that Eq. (51) recoves Eq. (31) when $\gamma\to0$.

It is natural to assume that the total level width can be broken into three components,

$$\gamma = \gamma_R + \gamma_L + \gamma_0, \tag{52}$$

where γ_0 represents the broadening caused by effects other than the leakage of charge through the leads.

6.2 Adding spin

To add spin, we split the configuration where the molecule level is occupied into two ($\uparrow$, $\downarrow$), resulting in a total of three configurations: $i=0,\uparrow,\downarrow$ (we forbid double occupancy by assuming that the charging energy E_c is a very large energy scale, namely, $E_c \gg k_BT, eV, |\varepsilon_1|$). Let us assume that the molecular level is spin degenerate. Then, the total current through the left lead is given by the expression

$$I_L = -e\,[P_0\,(\Gamma^L_{0\to\uparrow}+\Gamma^L_{0\to\downarrow}) - P_\uparrow\,\Gamma^L_{\uparrow\to0} - P_\downarrow\,\Gamma^L_{\downarrow\to0}]. \tag{53}$$

The rate equations are

$$\frac{dP_0}{dt} = -P_0\,(\Gamma_{0\to\uparrow}+\Gamma_{0\to\downarrow}) + P_\uparrow\,\Gamma_{\uparrow\to0} + P_\downarrow\,\Gamma_{\downarrow\to0}, \tag{54}$$

$$\frac{dP_\uparrow}{dt} = -P_\uparrow\,\Gamma_{\uparrow\to0} + P_0\,\Gamma_{0\to\uparrow}, \tag{55}$$

$$\frac{dP_\downarrow}{dt} = -P_\downarrow\,\Gamma_{\downarrow\to0} + P_0\,\Gamma_{0\to\downarrow}. \tag{56}$$

Solving for the steady state yields

$$P_0 = \frac{\Gamma_{\uparrow\to 0}\,\Gamma_{\downarrow\to 0}}{\Gamma_{\uparrow\to 0}\,\Gamma_{\downarrow\to 0} + \Gamma_{0\to\uparrow}\,\Gamma_{\downarrow\to 0} + \Gamma_{0\to\downarrow}\,\Gamma_{\uparrow\to 0}}, \tag{57}$$

$$P_\uparrow = \frac{\Gamma_{0\to\uparrow}\,\Gamma_{\downarrow\to 0}}{\Gamma_{\uparrow\to 0}\,\Gamma_{\downarrow\to 0} + \Gamma_{0\to\uparrow}\,\Gamma_{\downarrow\to 0} + \Gamma_{0\to\downarrow}\,\Gamma_{\uparrow\to 0}}, \tag{58}$$

and

$$P_\downarrow = \frac{\Gamma_{0\to\downarrow}\,\Gamma_{\uparrow\to 0}}{\Gamma_{\uparrow\to 0}\,\Gamma_{\downarrow\to 0} + \Gamma_{0\to\uparrow}\,\Gamma_{\downarrow\to 0} + \Gamma_{0\to\downarrow}\,\Gamma_{\uparrow\to 0}}. \tag{59}$$

Assuming spin degeneracy in the leads, we find

$$\Gamma^l_{0\to\uparrow} = \Gamma^l_{0\to\downarrow} = (\gamma_l/\hbar)\int d\varepsilon\, D_1(\varepsilon)\, f\left(\frac{E_1 - E_0 - \mu_l}{k_B T}\right) \equiv \Gamma^l_{0\to 1}, l = L, R, \tag{60}$$

and

$$\Gamma^l_{\uparrow\to 0} = \Gamma^l_{\downarrow\to 0} = (\gamma_l/\hbar)\int d\varepsilon\, D_1(\varepsilon)\left[1 - f\left(\frac{E_1 - E_0 - \mu_l}{k_B T}\right)\right] \equiv \Gamma^l_{1\to 0}, l = L, R. \tag{61}$$

Therefore,

$$\Gamma_{0\to\uparrow} = \Gamma_{0\to\downarrow} = \int d\varepsilon D_1(\varepsilon)[\gamma_R\, f_R(\varepsilon) + \gamma_L\, f_L\,(\varepsilon)] \equiv \Gamma_{0\to 1} \tag{62}$$

and

$$\begin{aligned}
\Gamma_{\uparrow\to 0} = \Gamma_{\downarrow\to 0} &= \int d\varepsilon D_1(\varepsilon)[\gamma_R + \gamma_L - \gamma_R\, f_R(\varepsilon) - \gamma_L\, f_L\,(\varepsilon)]/\hbar && (63)\\
&= (\gamma_R + \gamma_L)/\hbar - \int d\varepsilon\, D_1(\varepsilon)[\gamma_R\, f_R(\varepsilon) + \gamma_L\, f_L\,(\varepsilon)]/\hbar && (64)\\
&= (\gamma_R + \gamma_L)/\hbar - \Gamma_{0\to 1} \equiv \Gamma_{1\to 0}, && (65)
\end{aligned}$$

where we introduced

$$f_l(\varepsilon) = f\left(\frac{E_1 - E_0 - \mu_l}{k_B T}\right). \tag{66}$$

Notice that rates and probabilities do not depend on spin. Thus, we can recast the problem in terms of P_0 and $P_1 = P_\uparrow + P_\downarrow$. Then, we find

$$P_0 = \frac{\Gamma_{1\to 0}}{\Gamma_{1\to 0} + 2\Gamma_{0\to 1}} \tag{67}$$

and

$$P_1 = \frac{2\Gamma_{0\to 1}}{\Gamma_{1\to 0} + 2\Gamma_{0\to 1}}. \tag{68}$$

Plugging them into the expression for the current, we get

$$\begin{aligned}
I_L &= -e\,(2P_0\,\Gamma^L_{0\to 1} - P_1\,\Gamma^L_{1\to 0}) && (69)\\
&= -2e\,\frac{\Gamma_{1\to 0}\,\Gamma^L_{0\to 1} - \Gamma_{0\to 1}\Gamma^L_{1\to 0}}{\Gamma_{1\to 0} + 2\Gamma_{0\to 1}} && (70)\\
&= -2e\,\frac{\Gamma^R_{1\to 0}\,\Gamma^L_{0\to 1} - \Gamma^R_{0\to 1}\Gamma^L_{1\to 0}}{(\gamma_R + \gamma_L)/\hbar + \Gamma_{0\to 1}} && (71)\\
&= -2\,\frac{e}{h}\,\frac{\gamma_R\,\gamma_L}{(\gamma_R + \gamma_L) + \hbar\Gamma_{0\to 1}}\int d\varepsilon\,\frac{\gamma}{(\varepsilon - \varepsilon_1 + eV_g)^2 + (\gamma/2)^2}\left[f\left(\frac{\varepsilon - \mu_L}{k_B T}\right) - f\left(\frac{\varepsilon - \mu_R}{k_B T}\right)\right], && (72)
\end{aligned}$$

where

$$\Gamma_{0\to 1} = \int d\varepsilon\,\frac{\gamma}{(\varepsilon - \varepsilon_1 + eV_g)^2 + (\gamma/2)^2}\left[\gamma_R\, f\left(\frac{\varepsilon - \mu_L}{k_B T}\right) + \gamma_L\, f\left(\frac{\varepsilon - \mu_R}{k_B T}\right)\right]/\hbar. \tag{73}$$

Notice that because of the term $\Gamma_{0\to 1}$ in the denominator of the prefactor in Eq. (72), the expression for the current in the presence of spin is not exactly equal to twice that for the spinless case. However, if we are only interested in linear response, we can set $\mu_L = \mu_R = \mu$ in Eq. (73), in which case we obtain $\Gamma_{0\to 1} \approx (\gamma_R + \gamma_L)/\hbar$, provided that $\varepsilon_1 - eV_g < \mu$ (namely, when the energy level is brought below the Fermi energy in the leads). Then, the factor of 2 is approximately cancelled and we recover the expression for the spinless current. The current for the spinfull case is only exactly equal to twice that for the spinless case when the charging energy in the molecule is zero (non-interacting limit), in which case conductance through the molecule is spin degenerate.

7 Exact solution of the single-level case (spinless)

It is possible to solve exactly the fully coherent single-level case by using the Keldysh non-equilibrium technique [7], or even scattering theory, since no many-body interactions are present [8]. The result is the following: the probability of the level to be occupied is equal to

$$P_1 = \sum_l \frac{\gamma_l}{\gamma_R + \gamma_L} \int \frac{d\varepsilon}{2\pi} f\left(\frac{\varepsilon - \mu_l}{k_B T}\right) \frac{\gamma}{(\varepsilon - \varepsilon_1 + eV_g)^2 + (\gamma/2)^2}, \tag{74}$$

where $\gamma = \gamma_R + \gamma_L$ (absence of any level broadening other than leakage of charge through the leads). The probability of the empty level configuration is $P_0 = 1 - P_1$. The expressions for the probabilities are identical to those obtained with the rate equations after the broadening of the energy level is incorporated.

The fact that the coherent and incoherent formulations yield the same results for the probabilities is not surprising. For single channel leads and a single level in the molecule, interference plays no role since there is only one conduction path. When the molecule has multiple independent paths for electrons to hop in and out, then the coherent and incoherent predictions depart, since interference between paths can result in enhancement or depletion of certain configuration occupations.

An expression for the current was derived by Jauho, Wingreen, and Meir [9] using the Keldysh Green's function technique. Their result is

$$I_L = -\frac{e}{h} \int d\varepsilon \frac{\gamma_R \gamma_L}{(\varepsilon - \varepsilon_1 + eV_g)^2 + (\gamma/2)^2} \left[f\left(\frac{\varepsilon - \mu_L}{k_B T}\right) - f\left(\frac{\varepsilon - \mu_R}{k_B T}\right) \right]. \tag{75}$$

Contrary to our previous derivation using rate equations, this expression fully takes into account coherence. Yet, Eq. (75) and Eq. (51) are identical, provided that we set $\gamma = \gamma_R + \gamma_L$ (namely, no level broadening other than that due leakage through the leads). To some extend this should come as a surprise, as the coherent transport formulation contains incoherent, sequential regime as a limit.

Notice that for the spinfull case, one simply need to insert a factor of 2 on the right-hand-side of Eq. (75).

Asymptotic limits for the current

Notice that

$$f\left(\frac{\varepsilon - \mu_L}{k_B T}\right) - f\left(\frac{\varepsilon - \mu_R}{k_B T}\right) = \frac{\sinh\left(\frac{eV_b}{2k_BT}\right)}{\cosh\left(\frac{\varepsilon + E_F}{k_BT}\right) + \cosh\left(\frac{eV_b}{2k_BT}\right)}, \tag{76}$$

where $eV_b = \mu_L - \mu_R$ and $E_F = (\mu_L + \mu_R)/2$. Defining $\varepsilon' = \varepsilon - \varepsilon_1 + eV_g$, we can then rewrite Eq. (75) as

$$I_L = -\frac{e}{\hbar} \frac{\gamma_R \gamma_L}{\gamma_R + \gamma_L} \int d\varepsilon' \, D(\varepsilon') \left[\frac{\sinh\left(\frac{eV_b}{2k_BT}\right)}{\cosh\left(\frac{\varepsilon' + \varepsilon_1 - eV_g + E_F}{k_BT}\right) + \cosh\left(\frac{eV_b}{2k_BT}\right)} \right], \tag{77}$$

where

$$D(\varepsilon') = \frac{1}{\pi} \frac{\gamma/2}{\varepsilon'^2 + (\gamma/2)^2}. \tag{78}$$

It is easy to show that

$$\int d\varepsilon' \, D(\varepsilon') = 1. \tag{79}$$

Without loss of generality, we can set $eV_g = E_F$. Thus, ε_1 becomes the position of the energy level with respect the Fermi energy in the leads at zero bias. Then,

$$I_L = -\frac{e}{\hbar}\frac{\gamma_R\gamma_L}{\gamma_R+\gamma_L}\sinh\left(\frac{eV_b}{2k_BT}\right)\int d\varepsilon' \, D(\varepsilon')\frac{1}{\cosh\left(\frac{\varepsilon'+\varepsilon_1}{k_BT}\right)+\cosh\left(\frac{eV_b}{2k_BT}\right)}. \tag{80}$$

Let us look at some asymptotic limits.

- $\gamma \ll e\,|V_b| \ll k_BT \ll |\varepsilon_1|$: Weak broadening, finite bias, large temperature.

$$I_L \approx -\frac{e}{\hbar}\frac{\gamma_R\gamma_L}{\gamma_R+\gamma_L}\left(\frac{eV_b}{2k_BT}\right)\int d\varepsilon' \, D(\varepsilon')\frac{1}{\cosh\left(\frac{\varepsilon'+\varepsilon_1}{k_BT}\right)+1} \tag{81}$$

$$\approx -\frac{e}{\hbar}\frac{\gamma_R\gamma_L}{\gamma_R+\gamma_L}\left(\frac{eV_b}{k_BT}\right)e^{-\varepsilon_1/k_BT}. \tag{82}$$

The current in this case shows an activation behavior, with the activation energy being the offset between the energy level in the molecule and the Fermi energy in the leads. Linear bias regime. On resonance, we find

$$I_L \approx -\frac{e}{\hbar}\frac{\gamma_R\gamma_L}{\gamma_R+\gamma_L}\left(\frac{eV_b}{k_BT}\right). \tag{83}$$

- $\gamma \ll |\epsilon_1| \ll k_BT \ll e\,|V_b|$: Weak broadening, intermediate temperature, large bias, nearly on resonance.

$$I_L \approx -\frac{e}{\hbar}\frac{\gamma_R\gamma_L}{\gamma_R+\gamma_L}\sinh\left(\frac{eV_b}{2k_BT}\right)\int d\varepsilon' \, D(\varepsilon')\frac{1}{1+\cosh\left(\frac{eV_b}{2k_BT}\right)} \tag{84}$$

$$\approx -\frac{e}{\hbar}\frac{\gamma_R\gamma_L}{\gamma_R+\gamma_L}. \tag{85}$$

The current is approximatelly temperature and bias independent (non-linear bias regime).

- $\gamma \ll k_BT \ll |\epsilon_1| \ll e|V_b|$: Similar to the previous case, more off-resonance.

$$I_L \approx -\frac{e}{\hbar}\frac{\gamma_R\gamma_L}{\gamma_R+\gamma_L}\frac{e^{e|V_b|/2k_BT}}{2}\int d\varepsilon' \, D(\varepsilon')\frac{1}{\cosh\left(\frac{\varepsilon'+\varepsilon_1}{k_BT}\right)+e^{e|V_b|/2k_BT}/2} \tag{86}$$

$$\approx -\frac{e}{\hbar}\frac{\gamma_R\gamma_L}{\gamma_R+\gamma_L}. \tag{87}$$

The current is again approximatelly temperature and bias independent.

- $\gamma \ll k_BT \ll e|V_b| < |\epsilon_1|$: Similar to the previous case, but even more off-resonance.

$$I_L \approx -\frac{e}{\hbar}\frac{\gamma_R\gamma_L}{\gamma_R+\gamma_L}\frac{e^{e|V_b|/2k_BT}}{2}\int d\varepsilon' \, D(\varepsilon')\frac{1}{\cosh\left(\frac{\varepsilon'+\varepsilon_1}{k_BT}\right)+e^{e|V_b|/2k_BT}/2} \tag{88}$$

$$\approx -\frac{e}{\hbar}\frac{\gamma_R\gamma_L}{\gamma_R+\gamma_L}e^{(e|V_b|/2-|\varepsilon_1|)/k_BT}. \tag{89}$$

The current shows activation behavior and is highly non-linear.

- $\gamma, e|V_b|, |\epsilon_1| \ll k_BT$: High-temperature regime.

$$I_L \approx -\frac{e}{\hbar}\frac{\gamma_R\gamma_L}{\gamma_R+\gamma_L}\left(\frac{eV_b}{2k_BT}\right)\int d\varepsilon' \, D(\varepsilon')\frac{1}{2} \tag{90}$$

$$\approx -\frac{e}{\hbar}\frac{\gamma_R\gamma_L}{\gamma_R+\gamma_L}\left(\frac{eV_b}{4k_BT}\right). \tag{91}$$

The current decreases with the inverse of the temperature (linear bias regime).

- $k_B T \ll \gamma$: Low-temperature regime; strong broadening.

$$I_L \approx -\frac{e}{\hbar}\frac{\gamma_R\gamma_L}{\gamma_R+\gamma_L}\int_{-\varepsilon_1-eV_b/2}^{-\varepsilon_1+eV_b/2} d\varepsilon'\, D(\varepsilon') \tag{92}$$

$$\approx -\frac{2e}{h}\frac{\gamma_R\gamma_L}{\gamma_R+\gamma_L}\left[\arctan\left(\frac{-\varepsilon_1+eV_b/2}{\gamma/2}\right)-\arctan\left(\frac{-\varepsilon_1-eV_b/2}{\gamma/2}\right)\right]. \tag{93}$$

Notice that Eq. (93) is the starting point of a well-known theoretical description of electronic transport in "soft" molecular electronics [10,11]. The current is temperature independent and becomes linear with the bias voltage when $e|V_b| \ll \gamma$:

$$I_L \approx -\frac{e^2}{h}\frac{4\gamma_R\gamma_L}{\gamma(\gamma_R+\gamma_L)}V_b. \tag{94}$$

8 Conclusions

Given that for single-channel, single-level conductance both fully coherent and sequentially incoheren approaches lead to the same expression for the current, we can conclude that the most general expression (at low bias) is given by

$$I_L = -\frac{e}{h}\frac{\gamma_R\gamma_L}{\gamma_R+\gamma_L}\int d\varepsilon \frac{\gamma}{(\varepsilon-\varepsilon_1+eV_g)^2+(\gamma/2)^2}\left[f\left(\frac{\varepsilon-\mu_L}{k_B T}\right)-f\left(\frac{\varepsilon-\mu_R}{k_B T}\right)\right], \tag{95}$$

where we allow the total level width to include some broadening due to energy relaxation mechanisms other than leakage through the leads, namely, $\gamma = \gamma_R + \gamma_L + \gamma_0$.

9 References

1. D. V. Averin and A. N. Korotkov, Zh. Eksp. Teor. Fiz. **97**, 1661 (1990) [*Influence of discrete energy spectrum on correlated electron-electron tunneling via a mezoscopic small metal granule*, Sov. Phys. JETP **70**, 937 (1990)].
2. J. H. Davis, S. Hershfield, P. Hyldgaard, and J. W. Wilkins, *Current and rate equations for resonant tunneling*, Phys. Rev. B **47**, 4603 (1992).
3. S. A. Gurvitz and Ya. S. Prager, *Microscopic derivation of rate equations for quantum transport*, Phys. Rev. B **53**, 15932 (1996).
4. H.-P. Breuer and F. Petruccione, *The Theory of Open Quantum Systems* (Oxford Press, New York, 2002).
5. L. P. Kadanoff and G. Baym, *Quantum Statistical Mechanics* (W. A. Benjamin, Reading, MA, 1962).
6. L. V. Keldysh, Zh. Eksp. Teor. Fiz. **47** 1515 (1964) [*Diagram technique for nonequilibrium processes*, Sov. Phys. JETP **20**, 1018 (1965)].
7. H. J. W. Haug and A.-P. Jauho, *Quantum Kinetics in Transport and Optics of Semiconductors*, 2nd edition (Springer, 2008).
8. J. Koch, F. von Oppen, and A. V. Andreev, *Theory of the Frank-Condon blockade regime*, Phys. Rev. B **74**, 205438 (2006).
9. A.-P. Jauho, N. S. Wingreen, and Y. Meir, *Time-dependent transport in interacting and noninteracting resonant-tunneling systems*, Phys. Rev. B **50**, 5528 (1994).
10. I. Baldea and H. Koppel, *Sources of negative differential resistance in electrical nanotransport*, Phys. Rev. B **81**, 193401 (2010).
11. I. Baldea, *Ambipolar transition voltage spectroscopy: Analytical results and experimental agreement*, Phys. Rev. B **85**, 035442 (2012).
